# Gene Expression of Protein Tyrosine Phosphatase 1B and Endoplasmic Reticulum Stress During Septic Shock

**DOI:** 10.3389/fmed.2019.00240

**Published:** 2019-11-01

**Authors:** Thomas Clavier, Steven Grangé, Thibaut Pressat-Laffouilhere, Emmanuel Besnier, Sylvanie Renet, Sylvain Fraineau, Pierre-Alain Thiebaut, Vincent Richard, Benoit Veber, Fabienne Tamion

**Affiliations:** ^1^Department of Anesthesiology and Critical Care, Rouen University Hospital, Rouen, France; ^2^Normandie Univ, UNIROUEN, INSERM U1096, FHU REMOD-VHF, Rouen, France; ^3^Department of Medical Critical Care, Rouen University Hospital, Rouen, France; ^4^Department of Biostatistics, Rouen University Hospital, Rouen, France

**Keywords:** protein tyrosine phosphatase, non-receptor type 1/metabolism, endoplasmic reticulum stress, sepsis, shock, endothelium, inflammation

## Abstract

**Introduction:** Protein Tyrosine Phosphatase 1B (PTP1B) and endoplasmic reticulum stress (ERS) are involved in the septic inflammatory response. Their inhibition is associated with improved survival in murine models of sepsis. The objective was to describe PTP1B and ERS expression during septic shock in human.

**Material and Methods:** Prospective study including patients admitted to intensive care unit (ICU) for septic shock. Blood samples were collected on days 1 (D1), 3 and 5 (D5). Quantitative PCR (performed from whole blood) evaluated the expression of genes coding for PTP1B (*PTPN1*) and key elements of ERS (GRP78, ATF6, CHOP) or for endothelial dysfunction-related markers (ICAM1 and ET1). We analyzed gene variation between D5 and D1, collected glycemic parameters, insulin resistance and organ failure was evaluated by Sequential Organ Failure Assessment (SOFA) score.

**Results:** We included 44 patients with a mean SAPS II 50 ± 16 and a mortality rate of 13.6%. Between D1 and D5, there was a significant decrease of *PTPN1* (*p* < 0.001) and *ATF6* (*p* < 0.001) expressions. Their variations of expression were correlated with SOFA variation (*PTPN1, r* = 0.35, CI 95% [0.05; 0.54], *p* = 0.03 and *ATF6, r* = 0.45 CI 95% [0.20; 0.65], *p* < 0.001). We did not find any correlation between *PTPN1* expression and insulin resistance or glycemic parameters. Between D1 and D5, *ATF6* and *PTPN1* expressions were correlated with that of *ET1*.

**Conclusions:** Our study has evaluated for the first time the expression of PTP1B and ERS in patients with septic shock, revealing that gene expression variation of *PTPN1* and *ATF6* are partly correlated with the evolution of septic organ failure and with endothelial dysfunction markers expression.

## Introduction

Sepsis-caused multiple organ dysfunction syndrome (MODS) remains a major cause of morbidity and mortality in intensive care units (ICU) (1). Endothelial injury plays a major role in the pathogenesis of organ dysfunction during sepsis: endothelial dysfunction leads to the breakdown of the microvascular barrier resulting in increased extravascular fluid, tissue edema and organ failure (2, 3).

Protein Tyrosine Phosphatase 1B (PTP1B), a phosphatase localized on the cytoplasmic side of the endoplasmic reticulum (ER), is involved in the negative regulation of many cell pathways such as response to insulin or endothelial nitric oxide (NO) production (4). Our team and others recently showed that pharmacological or genetic inhibition of PTP1B restores vascular relaxation and endothelial NO synthase phosphorylation in septic models, protects against cardiac inflammation and dysfunction, and reduces mortality, suggesting its potential interest for the development of sepsis therapies that target endothelial injury (5, 6). The septic inflammatory process also causes serious disturbances in the insulin signaling pathway (7, 8). We previously demonstrated that PTP1B gene (*Protein Tyrosine Phosphatase Non-Receptor Type 1* (*PTPN1*)) deletion significantly limits cecal ligation and puncture (CLP) -induced insulin resistance, improves insulin receptor signal transduction and reduces sepsis-induced endothelial dysfunction/impaired NO production (9). Multiple disturbances observed during sepsis can result in a dysfunction of the ER, leading to the accumulation of unfolded proteins within the lumen of the ER, known as ER stress (ERS) (10). Recent studies indicated that PTP1B and ERS are closely interlinked: ERS induces and activates PTP1B in skeletal muscle *via* the NF-κB pathway and PTP1B inhibition protects against ERS-induced cardiac dysfunction (11, 12). PTP1B plays a major role in the regulation of ERS in endothelial cell, and genetic or pharmacological inhibition of PTP1B improves endothelial dysfunction induced by ERS (13).

The defense against ERS mainly involves the unfolded protein response (UPR). UPR is initiated by chaperone [such as the 78 kDa Glucose Related Protein (GRP78)] binding to unfolded peptides, which relieves the basal inhibitory signal of signaling pathways of UPR including Protein Kinase RNA-like ER kinase [PERK, involving CCAAT/enhancer binding protein homologous protein (CHOP)] and Activating Transcription Factor 6 (ATF6) pathways (10, 14). Systemic inflammation is associated with the activation of ERS pathways which are strongly activated in murine models of sepsis (15–17). Treatment with 4-phenylbutyric acid (4BPA; an ERS inhibitor) decreased the tissue expression level of inflammatory cytokines, NF-κB activation and reduced organ dysfunction induced by bacterial lipopolysaccharide (LPS) (16). 4BPA also improved the mortality rate in a murine CLP model (18). In human, ERS is activated in the mononuclear cells of patients with acute lung injury and is involved in acute kidney injury (16, 19). Finally, it has been shown that ERS is associated with endothelial dysfunction and its inhibition improves endothelium-dependent relaxing function (13). Thus, modulation of ER stress *via* PTP1B inhibitors may be a promising approach to protect the endothelium in sepsis but to our knowledge no study has evaluated their expression in severe infections in humans.

The objectives of our study were therefore to explore the relationship between PTP1B and ERS gene expression and organ failure during septic shock in humans, and to describe their kinetics.

## Materials and Methods

### Patient Population

This prospective pilot study was carried out in the medical ICU of a tertiary care University Hospital. The study, conducted between December 2015 and April 2016 (N°2014-A00959-38), was approved by the ethics committee of Rouen University Hospital (n° CPP 02/017/2014) in accordance with the ethical standards of the Declaration of Helsinki and its later amendments. Written consent was provided prospectively by the authorized representatives and/or retrospectively by the patient.

Patients admitted to medical ICU with septic shock as defined by the 2013 surviving sepsis campaign guidelines were included (20). Exclusion criteria were age under 18 years or patient under tutorship, pregnancy/ breastfeeding, obesity defined by a body mass index ≥30 kg/m^2^, diabetes mellitus treated by oral and/or insulin therapy, and patient refusal. Each patient included in the protocol had a specific follow-up for the study during the first 5 days of their ICU stay (D1 to D5). The following parameters were evaluated: demographic characteristics [sex, age, Simplified Acute Physiology Score II (SAPS II)], insulin resistance measurement using the Homeostasis Model Assessment of Insulin Resistance score (HOMA-IR) on the first day (with insulin dosage at D1), daily maximal or minimal capillary glycemia, daily physiologic parameters, sepsis origin, daily Sequential Organ Failure Assessment (SOFA) score, daily diuresis, cumulative daily dose of norepinephrine and biological parameters (lactate, procalcitonin (PCT), glycemia) at the time of each sample.

### Objectives

The main objective of this study was to search for a relationship between whole blood *PTPN1* gene (coding for PTP1B) expression and SOFA score, by comparing the variation in *PTPN1* gene expression and the variation in SOFA score calculated between D1 and D5 (presented as a delta-SOFA score: SOFA D5-SOFA D1).

Secondary objectives were to study:

- The link between daily *PTPN1* expression and insulin resistance assessment (HOMA score) at D1, and the link between daily *PTPN1* expression and daily cumulative dose of insulin administered, daily blood glucose variability (evaluated via blood glucose meter), and venous glycemia at the time of sampling at D1, D3, and D5;
- The correlation between the expression of genes involved in UPR (*ATF6, CHOP, GRP78*, and the variation in SOFA score;
- The kinetics of *PTPN1* and UPR gene expression in patients between D1 and D5;
- The correlation between *PTPN1* and UPR gene expression and clinical or biological markers of sepsis severity: survival, norepinephrine infusion doses, daily diuresis, and SAPS II at D1;
- The correlation between *PTPN1* and UPR gene expression and the expression of the genes involved in endothelial dysfunction *ICAM1* (coding for Inter-Cellular Adhesion Molecule 1) and *ET1* (coding for Endothelin 1).

### Sample Collection and Analysis

In addition to the usual daily morning blood sample, a 2.5 ml sample was drawn using PAXgene tube (Quiagen, Hilden, Germany) in the morning at 8:00 a.m. at D1, D3, and D5. Blood samples were kept for a maximum of 72 h in the refrigerators of the ICU at −20°C and then were stored at −80°C until analysis. PAXgene sampling was stopped after ICU discharge (if occurring before D5).

### Ribonucleic Acid Extraction, Reverse Transcription, and Quantitative Polymerase Chain Reaction

Briefly, ribonucleic acid (RNA) extraction was performed using the commercial kit PAXgene® Blood RNA System kit (Quiagen, Hilden, Germany) according to the manufacturer's protocol. Before RNA elution, residual genomic deoxyribonucleic acid (DNA) was digested using RNase-Free DNase set (Quiagen, Hilden, Germany). The integrity and quantity of the total RNA were assessed with a Nanodrop 2000 device (Thermo Fisher Scientific, Waltham, MS, USA). Total RNAs were reverse transcribed into cDNA using M-MLV Reverse Transcriptase (Invitrogen, Carlsbad, CA, USA) according to manufacturer's instructions.

A quantitative polymerase chain reaction (qPCR) was performed for:

- The mRNA of genes coding for PTP1B (*PTPN1* gene) and for proteins involved in UPR: ATF6, CHOP, GRP78;
- The mRNA of genes coding for proteins associated with septic endothelial dysfunction: ICAM1 and ET1;
- The mRNA of the gene coding for succinate dehydrogenase complex flavoprotein subunit A (SDHA). As *SDHA* has been described as a pertinent housekeeping gene in humans with sepsis and as its cycle threshold (Ct) is close to the Ct of PTP1B and UPR genes in qPCR, it appeared as the best housekeeping gene for our work (21).

The genes that were amplified and the primers that were used are listed in Table 1. PCR reactions were performed on a LightCycler 480 instrument using the Fast-StartDNA Master SYBR Green I real-time PCR kit according to the manufacturer's instructions (Roche Molecular Biochemicals, Bâle, Switzerland). Thermocycling was performed in a final volume of 10 μl (including 2.5 μl of cDNA sample at 1.67.10^−3^ μg/μl diluted at 1:10) containing 0.5 μl of each required primers at a concentration of 10 μM. PCR was performed with an initial denaturation step of 10 min at 95°C, followed by 40 cycles of a touch-down PCR protocol (40 cycles at 95°C for 10 s, 60°C for 10 s, 72°C for 12 s and 1 melting cycle). Ct values were used for quantifying target gene expression relative to the housekeeping gene using the 2^−ΔCt^ method. Variation in gene expression was defined as the ratio of gene expression between D5 and D1 (D5/D1), e.g., resulting in a ratio of 2 if the expression doubled or a ratio of 0.5 if the expression halved at D5.

**Table 1:** Primers used for quantitative PCR.

**Gene name**	**Sense**	**Sequence (5^**′**^-3^**′**^)**
*SDHA*	Forward	GAGATGTGGTGTCTCGGTCCAT
	Reverse	GCTGTCTCTGAAATGCCAGGCA
*PTP1B*	Forward	TGTCTGGCTGATACCTGCCTCT
	Reverse	ATCAGCCCCATCCGAAACTTCC
*CHOP*	Forward	TCGCCGAGCTCTGATTGAC
	Reverse	CCCTGCGTATGTGGGATTGAG
*ATF6*	Forward	CCGCAGAAGGGGAGACACA
	Reverse	TCGGAGGTAAGGAGGAACTGACG
*GRP78*	Forward	CGAGGAGGAGGACAAGAAGG
	Reverse	CACCTTGAACGGCAAGAACT
*ET1*	Forward	GCCCTCCAGAGAGCGTTATG
	Reverse	AGACAGGCCCCGAAGGTCT
*ICAM1*	Forward	GGCCGGCCAGCTTATACAC
	Reverse	TAGACACTTGAGCTCGGGCA

*ATF6, Activating Transcription Factor 6; CHOP, DNA Damage Inducible Transcript 3; ET1, Endothelin 1; GRP78, Heat Shock 70 kDa Protein 5; ICAM1, Inter-Cellular Adhesion Molecule 1; PTPN1, Protein Tyrosine Phosphatase Non-Receptor Type 1; SDHA, succinate dehydrogenase complex flavoprotein subunit A*.

### Statistical Analysis

Quantitative values are expressed as mean with standard deviation (for demographic data) and confidence interval at 95% (for SOFA score and gene expression). Qualitative values are presented with absolute values and percentages. Gene expression comparisons between D1 and D5 and correlations were carried out after logarithmic transformation and are therefore described in this manner in the figures. The initial (D1) and the final (D5) levels of log transformed gene expression were compared with a Paired Student test. Among patients with a significant change in gene expression between D1 and D5, correlations between variations in log transformed gene expression were computed with the Pearson method, 95% confidence intervals were calculated with the Bootstrap method. Missing data were managed for the correlation analysis, the last-observation-carried-forward method was used when needed (when D5 values were missing, D3 values replaced it). The significance level was 0.05 for all the tests performed. All calculations and statistical analyses were conducted on R 3.4.2 statistical software.

## Results

### Clinical and Demographic Characteristics of Population

**Figure 1:**
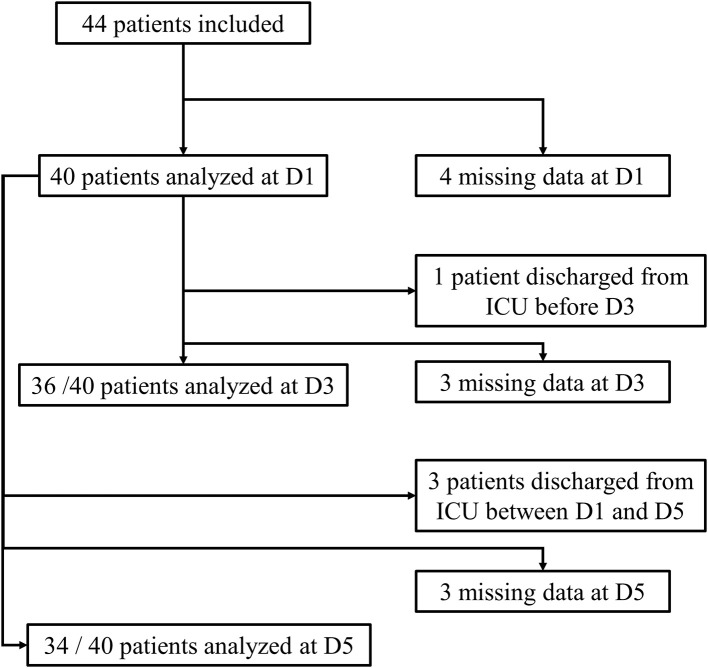
Flow-chart of the study.

**Figure 2:**
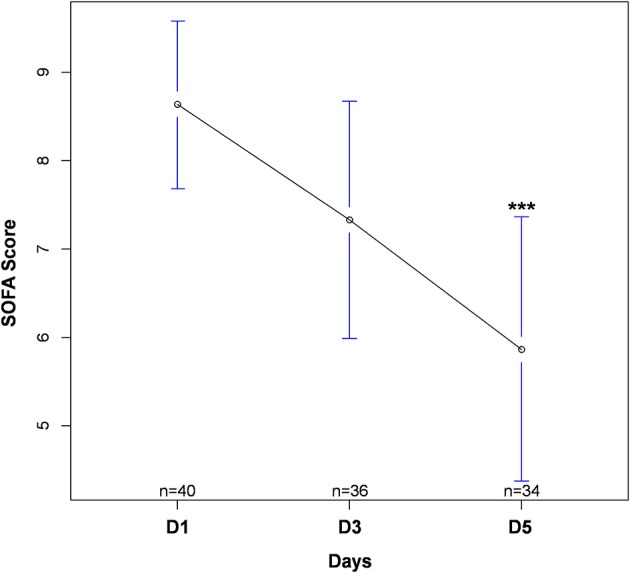
Evolution in SOFA score during intensive care unit stay. Values are expressed as mean with 95% confidence interval. Numbers (n) correspond to the number of patients still alive and in ICU at each time. ***p < 0.001 between D1 and D5. SOFA, Sequential Organ Failure Assessment.

Forty-four patients with septic shock were included in this study. Diagnoses at admission were: pneumonia (*n* = 26, 59%), urinary-tract infection (*n* = 10, 23%), intra-abdominal infection (*n* = 4, 9%), other (*n* = 4, 9%). Due to technical problems (RNA extraction difficulties) or lack of genetic material after extraction, some samples could not be analyzed. Four patients had no sample measurement on D1 and therefore could not be analyzed in regard to the main objective. In addition, some patients were discharged from ICU before D3 or before D5. Thus, of the 40 patients remaining on whom the analysis was conducted, 30 had all measurements at D1, D3, and D5, 4 had a measurement at D1 and D5 only and 6 had a measurement at D1 and D3 only. The study flow chart is presented in Figure 1. Between D1 and D5, there was a significant decrease in SOFA score (*cf*. Figure 2). The main demographic and clinical characteristics of patients are summarized in Table 2, the leucocyte cell count at each sampling time are summarized in Table 3.

**Table 2:** Main demographic and clinical characteristics of included patients.

Age (years)	65 ± 15
Sex-ratio (M/F)	2.7 (32/12)
SAPS II	50 ± 16
Length of stay in ICU (days)	11 ± 9
Mechanical ventilation	24 (54.5%)
Mortality at D28	6 (13.6%)

*Data are presented as mean with standard deviation or absolute value and percentage [n (%)]. SAPS II, Simplified Acute Physiology Score II*.

**Table 3:** Leucocyte cell count at each sampling time.

	**Day 1**	**Day 3**	**Day 5**
Leucocytes (G/l)	19.7 ± 12.3	15.2 ± 9.0	12.5 ± 11.5
Neutrophils granulocytes (G/l)	17.6 ± 11.6 (89.2%)	13.4 ± 8.2 (88.0%)	10.2 ± 8.8 (81.8%)
Eosinophils granulocytes (G/l)	0.1 ± 0.1 (0.5%)	0.1 ± 0.2 (0.7%)	0.2 ± 0.2 (1.3%)
Basophils granulocytes (G/l)	0.0 ± 0.0 (0%)	0.0 ± 0.0 (0%)	0.0 ± 0.0 (0%)
Lymphocytes (G/l)	1.1 ± 0.9 (5.6%)	0.9 ± 0.9 (6.0%)	1.1 ± 0.7 (8.6%)
Monocytes (G/l)	0.9 ± 0.7 (4.7%)	0.8 ± 0.5 (5.3%)	1.0 ± 0.9 (8.3%)

*Data are presented as mean with standard deviation, in brackets are the proportions of each population to the total number of leukocytes*.

### PTP1B Gene Expression

**Figure 3:**
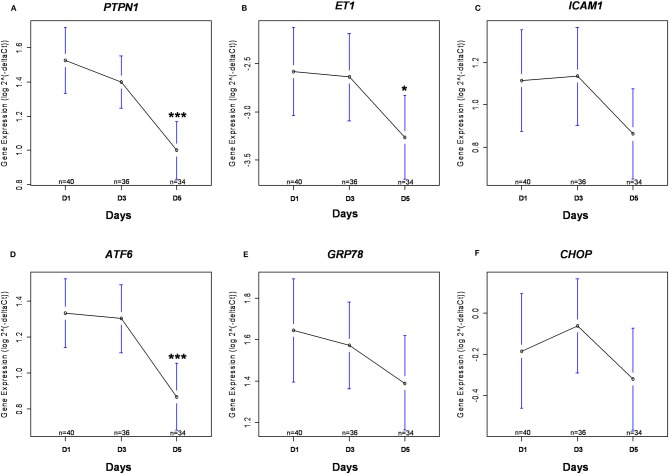
Kinetics of gene expression. Gene expression of *PTPN1* **(A)**, *ET1* **(B)**, *ICAM1* **(C)**, *ATF6* **(D)**, *GRP78* **(E)**, and *CHOP* **(F)** are presented after logarithmic transformation as mean with 95% confidence interval. The numbers (*n*) correspond to the number of patients still alive and in ICU at each time. *p < 0.05 between D1 and D5; ***p < 0.001 between D1 and D5. ATF6, Activating Transcription Factor 6; CHOP, DNA Damage Inducible Transcript 3; ET1, endothelin 1; GRP78, Heat Shock 70 kDa Protein 5; ICAM1, inter-cellular adhesion molecule 1; PTPN1, Protein Tyrosine Phosphatase Non-Receptor Type 1.

There was a statistically significant decrease in *PTPN1* expression between D1 and D5 (*cf*. Figure 3A). Concerning glycemic function, we did not find any statistically significant correlation between HOMA-IR and *PTPN1* expression at D1, or any correlation between *PTPN1* expression and daily dose of insulin, daily blood glucose variability and maximum daily blood glucose peak from D1 to D5 (*cf*. Table 4). No statistically significant correlation was observed between *PTPN1* expression and diuresis or cumulative daily dose of norepinephrine. There was no significant difference in *PTPN1* D1-D5 expression variations between deceased and surviving patients (*cf*. Table 5).

**Table 4:** Insulin resistance score, glycemic parameters, and their correlation with *PTPN1* expression during ICU stay.

	**Day 1**	**Day 3**	**Day 5**
HOMA-IR	4.7 ± 4.5 [0.03 (−0.32; 0.46)]	ND	ND
Daily dose of insulin (UI/day)	7.5 ± 21.6 [0.18 (−0.10; 0.39)]	13.4 ± 27.9 [0.06 (−0.14; 0.33)]	14.1 ± 25.0 [0.13 (−0.15; 0.32)]
Daily blood glucose variability (blood glucose meters; max–min) (mmol/l)	3.9 ± 3.4 [0.23 (−0.09; 0.50)]	2.6 ± 1.9 [−0.02 (−0.36; 0.32)]	2.7 ± 2.8 [0.24 (−0.15; 0.47)]
Venous glycemia at the time of sampling (mmol/l)	8.0 ± 3.1 [0.07 (−0.20; 0.34)]	7.8 ± 2.0 [−0.11 (−0.40; 0.19)]	6.9 ± 2.1 [0.20 (−0.16; 0.51)]

*Data are presented as mean with standard deviation. In brackets are the correlation coefficients of each value with PTPN1 expression of the corresponding day and its 95% confidence interval. HOMA-IR: homeostasis model assessment of insulin resistance score; ND, not done; PTPN1, Protein Tyrosine Phosphatase Non-Receptor Type 1*.

**Table 5:** Comparison of gene expression variations between deceased and surviving patients.

	**Surviving patients (*n* = 35)**	**Deceased patients (*n* = 6)**	***p***
SAPS II	48 ± 15	64 ± 7	0.01
Age (years)	65 ± 15	61 ± 14	0.41
Delta-SOFA score	−3.2 ± 3	−1.8 ± 4	0.56
*PTPN1* expression variation	−0.49 ± 0.70	−0.36 ± 0.49	0.42
*ET1* expression variation	−0.67 ± 1.5	0.21 ± 1.9	0.36
*ATF6* expression variation	−0.46 ± 0.60	−0.14 ± 0.65	0.25

*Only genes that had a significant variation of expression between D1 and D5 are presented. Data are presented as mean with standard deviation. Gene expression variations are presented after logarithmic transformation (log(ratioD5/D1)) and variations in SOFA are presented as a delta-SOFA score (= SOFA D5–SOFA D1). ATF6, Activating Transcription Factor 6; ET1, endothelin 1; PTPN1, Protein Tyrosine Phosphatase Non-Receptor Type 1; SAPS II, Simplified Acute Physiology Score II; SOFA, Sequential Organ Failure Assessment*.

### Endothelial Dysfunction Markers and UPR Gene Expression Kinetics

Between D1 and D5, there was a significant decrease in *ET1* expression (*cf*. Figure 3B), and the expression of *ICAM1* appeared to decrease gradually although no significant difference was found (*cf*. Figure 3C). Between D1 and D5, there was a significant decrease in *ATF6* expression (*cf*. Figure 3D), *GRP78* expression appeared to decrease gradually although no significant difference was found (*cf*. Figure 3E) and *CHOP* expression was stable over time (*cf*. Figure 3F).

No statistically significant correlation was observed between *ATF6* expression and daily diuresis or cumulative daily dose of norepinephrine. There was no significant difference in *ATF6* and *ET1* D1-D5 expression variations between deceased and surviving patients (*cf*. Table 5).

### Correlation Between *PTPN1* and *ATF6* Expressions and SOFA Score or Survival

**Figure 4:**
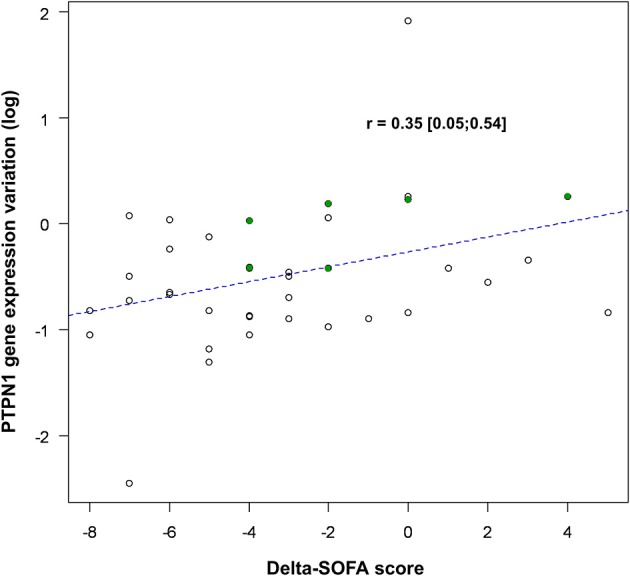
Correlation between variations in *PTPN1* expression and SOFA score. *PTPN1* expression variation is presented after logarithmic transformation [log(ratioD5/D1)] and variations in SOFA are presented as a delta-SOFA score (= SOFA D5–SOFA D1), showing a decrease of *PTPN1* expression when the SOFA decrease between D1 and D5. The points highlighted in green correspond to the patients for whom the results of D3 have been taken into account. PTPN1, Protein Tyrosine Phosphatase Non-Receptor Type 1; SOFA, Sequential Organ Failure Assessment.

**Figure 5:**
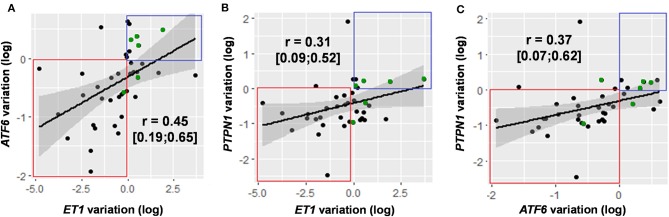
Correlation of variations in the expression of genes with significant variation in expression over time: *ET1/ATF6* **(A)**, *ET1/PTPN1* **(B)**, and *ATF6/PTPN1* **(C)**. Gene expression variations are presented after logarithmic transformation [log(ratioD5/D1)]. For each gene pair, data are presented in the form of a correlation coefficient with 95% confidence interval and in graphical form with regression line. Decreased expression of the two genes studied is framed in red, increased expression of the two genes studied is framed in blue. The points highlighted in green correspond to the patients for whom the results of D3 have been taken into account. ATF6, Activating Transcription Factor 6; ET1, endothelin 1; PTPN1, Protein Tyrosine Phosphatase Non-Receptor Type 1.

Between D1 and D5, variations in *PTPN1* expression and SOFA score were positively correlated (*r* = 0.35, CI 95% [0.05; 0.54]; *p* = 0.03; *cf*. Figure 4); *PTPN1* expression increased by 7% (CI 95% [1%; 14%]) per point of SOFA. *PTPN1* expression ratio was correlated with *ET1* (*r* = 0.31 CI 95% [0.09; 0.52], *p* = 0.008; *cf*. Figure 5).

Between D1 and D5, variations in *ATF6* expression and SOFA score were positively correlated (*r* = 0.45 CI 95% [0.20; 0.65], *p* < 0.001); *ATF6* expression increased by 9% (CI 95% [3%; 15%]) per point of SOFA. Between D1 and D5, *ATF6* expression was also positively correlated with *ET1* (*r* = 0.45 CI 95% [0.19; 0.65], *p* < 0.001) and with *PTPN1* (*r* = 0.37 CI 95% [0.07; 0.62], *p* = 0.02; *cf*. Figure 5).

## Discussion

In regard to recent literature, our finding that *PTPN1* and UPR genes are expressed in the plasma of patients with septic shock and that their expression shows significant variations suggests that these components may be involved in the pathophysiology of sepsis. To our knowledge, PTP1B and UPR have never been studied in patients with septic shock. We found that the variation in *PTPN1* expression between D1 and D5 was significantly positively correlated with the variation in SOFA score (as was *ATF6* expression). Additionally, *PTPN1* and *ATF6* expressions were correlated with *ET1* which codes for a protein associated with septic endothelial dysfunction.

### PTP1B Gene Expression

PTP1B is implicated in a wide range of cellular functions. PTP1B has a prominent role in the metabolism, through the regulation of insulin signaling, leading to the development of PTP1B inhibitors for the treatment of type 2 diabetes or obesity. However, the role of PTP1B in regulating immune signaling is also emerging in infectious diseases (22). Recently, it has been demonstrated that, in a *P. aeruginosa* infection model, PTP1B-deficient mice displayed enhanced bacterial clearance and reduced disease scores, suggesting that PTP1B might have a deleterious role in the host defense against bacterial infections (23). Moreover, it has been shown that, in a rat model of sepsis, PTP1B induced brain mitochondrial dysfunction associated with overproduction of reactive oxygen species (24). In animal models, genetic or pharmacological inhibition of PTP1B enhanced endothelial cell proliferation and NO production, and could also reverse septic endothelial dysfunction (5, 6, 9, 25). We have shown a correlation between variations in SOFA score and *PTPN1* expression. However, the design of our work does not allow us to establish a causal link between organ failure assessed by SOFA score and *PTPN1* expression. We did not find any link between markers of initial severity (SAPS II) and *PTPN1* expression at D1. It is possible that, in view of the delay between gene expression and effective protein synthesis, organ failure at one time may be related to the expression of *PTPN1* in the previous hours, which would explain the correlation found for the global variation in organ failure but not for failure at a given time.

Inflammation can directly contribute to insulin resistance by disrupting the insulin signaling pathway, in part *via* PTP1B activation. In a previous experimental study, we demonstrated that PTP1B gene deletion significantly limited CLP-induced insulin resistance, improved AMP-activated protein kinase signaling pathway and Glucose Transporter 4 translocation, and decreased inflammation (9). In the present work, we did not find any link between *PTPN1* levels and HOMA-IR index, glycemia, glycemia variation or insulin consumption. A previous work described a strong correlation between insulin resistance evaluated through HOMA-IR index and plasma PTP1B level in a population of patients with polycystic ovarian syndrome (26). Moreover, PTP1B inhibition significantly decreased HOMA-IR index in obese and diabetic mice suggesting a direct link between PTP1B level and HOMA-IR (27). There are two reports on the use of HOMA-IR index in ICU patients but both studies have limitations with great variance and the use of HOMA-IR is controversial in ICU (28, 29). In our work, the conditions for measuring insulin levels using HOMA-IR were not standardized for insulin treatment at the time of sampling. In regard to this strong bias, to the results of previous study on the link between PTP1B and insulin resistance, and to controversies on the efficiency of HOMA-IR to assess insulin resistance in ICU patients, we should be cautious and not conclude the absence of a link between PTP1B levels and insulin resistance in septic patients.

### UPR Gene Expression

We have shown a correlation between variations in SOFA score and *ATF6* expression. ATF6 upregulates many protective genes and downregulates many potentially damaging genes (30, 31). Previous studies have shown that ATF6 activation in cardiac myocytes protects the heart from ischemic damage, while inhibiting ATF6 has the opposite effect (32). Since ATF6 is activated by ischemia but inactivated upon reperfusion, we could consider that organ ischemia related to the disequilibrium of the oxygen consumption/supply balance during sepsis is associated with the expression of ATF6 (32). The improvement in organ dysfunction could be associated with a decrease in ATF6 linked to organ reperfusion. We did not show any significant variation in the other two UPR genes studied. *GRP78* seemed to decrease during the first 5 days and it is possible that a lack of power prevented us from showing a significant decrease. It is also possible that, among UPR pathways, ATF6 is the most intensely involved pathway during sepsis but additional experiments should be carried out to study this hypothesis before drawing any conclusion. Previous studies have shown a beneficial effect of ERS inhibition on survival and MODS, which suggest the involvement of ERS in critical illness-induced MODS (16, 18, 33). Finally, expression of *ATF6, PTPN1*, and *ET1* tended to decrease more in surviving patients than in deceased patients (although this variation did not appear significant). However, this work was not designed to reveal a difference in mortality according to gene expression variations, but future work should analyze this point.

### PTP1B/UPR Gene Expression and Endothelial Dysfunction

Endothelial dysfunction is considered as a precursor of tissue damage and end-organ dysfunction (34, 35). It has been shown that ET1 levels are significantly higher in non-survivors than in survivors and are correlated with organ dysfunction and mortality (2, 36, 37). We found that a variation in *PTPN1* expression was correlated with a variation in *ET1* expression. PTP1B is involved in endothelial dysfunction and it is known that PTP1B inhibition reduces sepsis-induced endothelial dysfunction and impaired NO production (9). There is no work studying the link between ET1 and PTP1B but, more than a direct link between these two markers, since ET1 is a marker of septic endothelial lesion, it is likely that the observed correlation could reflect the relationship between *PTPN1* expression and septic endothelial dysfunction. As it has been demonstrated that LPS-induced *ET1* expression occurs via an NF-κB-dependent pathway and, as ERS induces NF-κB, we can hypothesize that the correlation between *ATF6* and *ET1* expression involves, at least in part, the NF-κB pathway (16, 38). ERS induces endothelial dysfunction *in vivo* and we found that the variation in *ATF6* expression was correlated with the variation in endothelial dysfunction marker. We can thus hypothesize that ERS may be one of the numerous triggering factors of endothelial dysfunction during sepsis in human (13, 39). Finally, we found no significant difference for *ATF6, ET1*, and *PTPN1* variations between surviving and deceased groups. However, given the very small size of our cohort with only 6 deaths, it seems difficult to definitely conclude that there is no association between mortality and expression of these genes.

### Limitations

Our work has several major limitations. It is a pilot physiologic study with a limited number of patients without a control group. We included patients with several infectious sources while related animal studies were conducted in standardized models. It is thus possible that *PTPN1* and UPR genes have different expressions and kinetics depending on the kind of infection but, given the limited number of patients, it did not appear reasonable to compare patients according to their infectious source. We used the criteria of the 2013 surviving sepsis campaign to include patients. This work was designed before the change in sepsis diagnostic criteria, which explains why SEPSIS-3 criteria were not used here. Our mortality rate was quite low for a cohort of septic shock patients but it remained within the low mortality rates described in some recent works (15–20%) (40, 41). In addition, we have included many urinary sepsis whose prognosis is much better than sepsis from other sources (42). These combined factors probably explain the low mortality rate observed.

Our work focused on gene expression in the whole blood but we did not perform a direct protein assay. It is known that, in human muscle, *PTPN1* expression is related to PTP1B protein level and that the increase in *ATF6* mRNA in peripheral blood mononuclear cells is correlated with ATF6 protein synthesis (43, 44). These data suggest that, for PTP1B and ATF6, gene expression is a good indicator of protein synthesis in humans. This work was conducted in ICU patients with a limited access to tissue, and with the logistical necessity of taking samples during several days (including weekends and/or on-call periods). As most of the proteins studied in our work cannot be measured in blood without complex cell isolation techniques, RNA quantification appeared to be the best compromise. However, it seems important to consider a future work measuring ERS proteins and ERS gene expression at the same time points in septic patients to better specify their correlation in this specific population. As the RNA expression on whole blood measured using the Paxgen tubes is strongly correlated with the RNA expression in circulating leucocytes, we can assume that we detected variations in leucocyte gene expression (45). As it is known that UPR play a crucial role in immune cells, including differentiation, immune activation, antibody production and cytokine expression, it seemed relevant to study the leucocyte expression of UPR genes (46, 47). However, we may have missed a potentially greater variation in gene expression in tissues and organs, as observed in animal models (16, 18, 33). It is also important to note that several studies have focused on the interest of a genomic approach to classify sepsis according to its severity or prognosis (48–50). These genotypic studies have so far failed to adequately discriminate sepsis from non-septic inflammatory syndrome and have shown no link between organ failure or clinical evolution and genomic expression profile. Recent data suggest that, at the level of leucocyte transcriptome, alterations in the expression of classical inflammatory and anti-inflammatory as well as adaptive immunity genes occur simultaneously, not sequentially after severe injury (49). Thus, variations of many genes during septic shock shifts in a same manner over time (up- or down-regulation), and it appears difficult to analyze the impact of a specific gene expression variation independently of others. The genomic study during sepsis therefore remains an analysis with clear limitations, limiting the final interpretation of our results. Given the existence of several open genomic datasets performed in whole blood of septic patients which come from genome wide association studies, it also appears interesting to consider a future work on these database to analyze all the genes involved in endothelial dysfunction, PTP1B regulation and ERS (48).

## Conclusion

We have described for the first time PTP1B and ERS expression in patients with septic shock. We have shown that PTP1B and ATF6 might be correlated with organ failure (SOFA score) and endothelial dysfunction (*ET1* expression). The recent identification of PTP1B as a novel negative regulator of host defense against sepsis may have potential therapeutic implications. However, further studies are needed to better understand PTP1B and ERS implication during sepsis in humans.

## Data Availability Statement

The raw data supporting the conclusions of this manuscript will be made available by the authors, without undue reservation, to any qualified researcher.

## Ethics Statement

The studies involving human participants were reviewed and approved by ethics committee of Rouen University Hospital (n° CPP 02/017/2014). The patients/participants provided their written informed consent to participate in this study.

## Author Contributions

TC was involved in acquisition of data, in analysis and interpretation of data, and in manuscript draft. SG was involved in acquisition of data, in study design, and in interpretation of data. TP-L was involved in statistical analysis, in interpretation of data, and in manuscript draft. EB was involved in the study conception and design, in acquisition of data, and in manuscript revision. SR was involved in acquisition of data and in analysis and interpretation of data. SF and P-AT were involved in analysis and interpretation of data and in manuscript revision. VR, BV, and FT were involved in the study conception and design, in analysis and interpretation of data, and in manuscript revision. All authors contributed to manuscript revision, read, and approved the submitted version.

### Conflict of Interest

The authors declare that the research was conducted in the absence of any commercial or financial relationships that could be construed as a potential conflict of interest.

## Abbreviations

4BPA: 4-phenylbutyric acid
ATF6: Activating Transcription Factor 6
CHOP: CCAAT/enhancer binding protein homologous protein
CLP: Cecal ligation and puncture
Ct: Cycle threshold
CHOP: DNA Damage Inducible Transcript 3 (gene coding for CHOP)
DNA: Deoxyribonucleic acid
ET1: Endothelin 1
ER: Endoplasmic reticulum
ERS: Endoplasmic reticulum stress
GRP78: 78 kDa Glucose Related Protein
HOMA-IR: Homeostasis Model Assessment of Insulin Resistance
GRP78: Heat Shock 70 kDa Protein 5
ICAM1: Inter-cellular adhesion molecule 1
ICU: Intensive care units
IRE1: Inositol-Requiring Enzyme 1 alpha
LPS: Lipopolysaccharide
MODS: Multiple organ failure
NF-κB: Nuclear Factor-Kappa B
NO: nitric oxide
PCR: Polymerase chain reaction
PCT: Procalcitonin
PERK: Protein Kinase RNA-like ER kinase
PTP1B: Protein Tyrosine Phosphatase 1B
PTPN1: Protein Tyrosine Phosphatase Non-Receptor Type 1 (gene coding for PTP1B)
RNA: Ribonucleic acid
SAPS II: Simplified Acute Physiology Score II
SDHA: Succinate dehydrogenase complex flavoprotein subunit A
SOFA: Sequential Organ Failure Assessment
UPR: Unfolded protein response.
